# Layer-Oriented Lateral Superior Approach for Robotic Right Hemicolectomy: A Novel Embryology-Based Strategy and Initial Clinical Outcomes

**DOI:** 10.3390/jcm15208016

**Published:** 2026-10-16

**Authors:** Tetsu Yamamoto, Takahito Taniura, Kazunari Ishitobi, Keisuke Inoue, Shunsuke Kaji, Takayuki Tanaka, Takeshi Matsubara, Masaaki Hidaka

**Affiliations:** Department of Digestive and General Surgery, Faculty of Medicine, Shimane University, 89-1 Enya, Izumo 693-8501, Shimane, Japan; taniura@med.shimane-u.ac.jp (T.T.); stonefly@med.shimane-u.ac.jp (K.I.); inoue.keisuke@med.shimane-u.ac.jp (K.I.); s.kaji@med.shimane-u.ac.jp (S.K.); gapperu197675@gmail.com (T.T.); nanadai@med.shimane-u.ac.jp (T.M.); mahidaka@med.shimane-u.ac.jp (M.H.)

**Keywords:** colorectal cancer, complete mesocolic excision, robotic surgery, superior approach, embryological layers

## Abstract

**Background:** Complete mesocolic excision (CME) with D3 lymphadenectomy is an oncologically established procedure for right-sided colon cancer; however, the optimal dissection plane and surgical approach remain controversial. We developed a novel layer-oriented lateral superior approach (LSA) that enables the intentional, depth-tailored selection of embryological dissection planes in robotic right hemicolectomy. **Methods:** This retrospective single-center study included consecutive patients who underwent robotic right hemicolectomy using the LSA between June 2023 and May 2025. The dissection plane was selected according to tumor depth (≤T2 vs. ≥T3). Perioperative and short-term oncological outcomes were evaluated. **Results:** Thirty-six patients were included. The median operative time was 289 min, and the median blood loss was 10 mL. No intraoperative complications or conversions occurred. Postoperative complications were observed in 6 patients (16.7%), all ≤Clavien–Dindo grade II. The median number of harvested lymph nodes was 31, and main lymph node metastases around the middle colic artery were identified in three patients. Among patients who underwent curative-intent resection (stage I–III), the median follow-up was 16.9 months. The estimated 2-year disease-free and overall survival rates were 92.4% and 84.2%, respectively. **Conclusions:** The LSA was feasible and safe in this initial series and provides a novel layer-oriented strategy for robotic CME.

## 1. Introduction

Colorectal cancer is the third most commonly diagnosed malignancy and the second leading cause of cancer-related mortality worldwide [1]. Right-sided colon cancer accounts for approximately 40% of all colon cancers and is associated with a poorer prognosis and higher postoperative morbidity compared with left-sided disease [2]. Establishing a safe and oncologically reliable surgical strategy for right-sided colon cancer therefore remains a critical clinical challenge [3].

Surgical concepts differ between Eastern and Western countries: D3 lymphadenectomy with central vascular ligation (CVL) is widely performed in Eastern practice [4,5], whereas Western surgery emphasizes complete mesocolic excision (CME) based on embryological principles [6]. Despite differences in terminology, both strategies aim to achieve en bloc resection of the mesocolon along defined fascial planes with central vascular control [7,8]. However, the optimal anatomical plane for right-sided colon cancer remains controversial due to the multilayered structure of the retrocolonic fascia (comprising multiple layers such as the mesocolon, parietal peritoneal fascia, pre-renal fascia, and pre-duodenal pancreatic fascia, which provide multiple potential surgical planes) and the lack of uniformity in anatomical terminology [9,10,11,12].

Recent anatomical studies have clarified the complex fascial relationships surrounding the duodenum and ascending colon, suggesting that the appropriate dissection plane should be selected according to tumor depth [6]. Precise identification of these embryological layers is therefore essential for achieving oncologically sound CME while preserving mesocolic integrity.

Various approaches, including lateral, medial-to-lateral, and cranial techniques, have been described for right hemicolectomy [13,14,15]. Although robotic surgery offers articulated instruments and stable three-dimensional visualization, existing robotic approaches primarily focus on vascular exposure rather than intentional selection of embryological dissection planes [16]. To our knowledge, no robotic approach has been described that explicitly incorporates depth-tailored selection of embryological layers as a core surgical principle.

We therefore developed a layer-oriented lateral superior approach (LSA) for robotic right hemicolectomy. This technique enables early identification of key fascial planes and central vessels, enabling secure CME with CVL and flexible determination of the medial margin for lymph node dissection. The aim of this study was to describe the surgical concept of the LSA and to evaluate its safety and short-term oncological outcomes.

## 2. Materials and Methods

### 2.1. Patients

This retrospective single-center study included consecutive patients with right-sided colon cancer who underwent robotic right hemicolectomy using the LSA between June 2023 and May 2025 at Shimane University Hospital. Patients with histologically confirmed adenocarcinoma of the cecum, ascending colon, or hepatic flexure were eligible. Patients undergoing emergency surgery, combined major organ resection, or conversion to open surgery were excluded. Clinicopathological data, including perioperative outcomes and pathological findings, were collected from a prospectively maintained database. Tumor staging was determined according to the tumor–node–metastasis (TNM) classification (8th edition). All consecutive patients who underwent robotic right hemicolectomy during the study period were treated using the lateral superior approach, and no alternative robotic approach was employed.

### 2.2. Surgical Concept and Standardization of LSA

The LSA is a layer-oriented approach designed to enable intentional selection of the dissection plane based on embryological anatomy. To minimize operator-dependent variability, the surgical procedure was standardized with predefined key steps, including: lateral-to-medial mobilization with early identification of the retroperitoneal fascial layers, exposure of the superior mesenteric vessels from a cranial approach, and central vascular ligation with D3 lymphadenectomy. The dissection plane was selected according to tumor depth using predefined criteria as follows:

(1) For tumors ≤ T2, dissection was performed superficial to the parietal peritoneal fascia.
(2) For tumors ≥ T3, the dissection plane was extended posteriorly toward the anterior renal fascia to ensure an adequate radial margin.

Preoperative tumor depth was assessed using contrast-enhanced computed tomography (CT), colonoscopy, and double-contrast barium enema findings. The planned dissection plane was confirmed intraoperatively according to the anatomical findings.

### 2.3. Operative Technique

**Figure 1:**
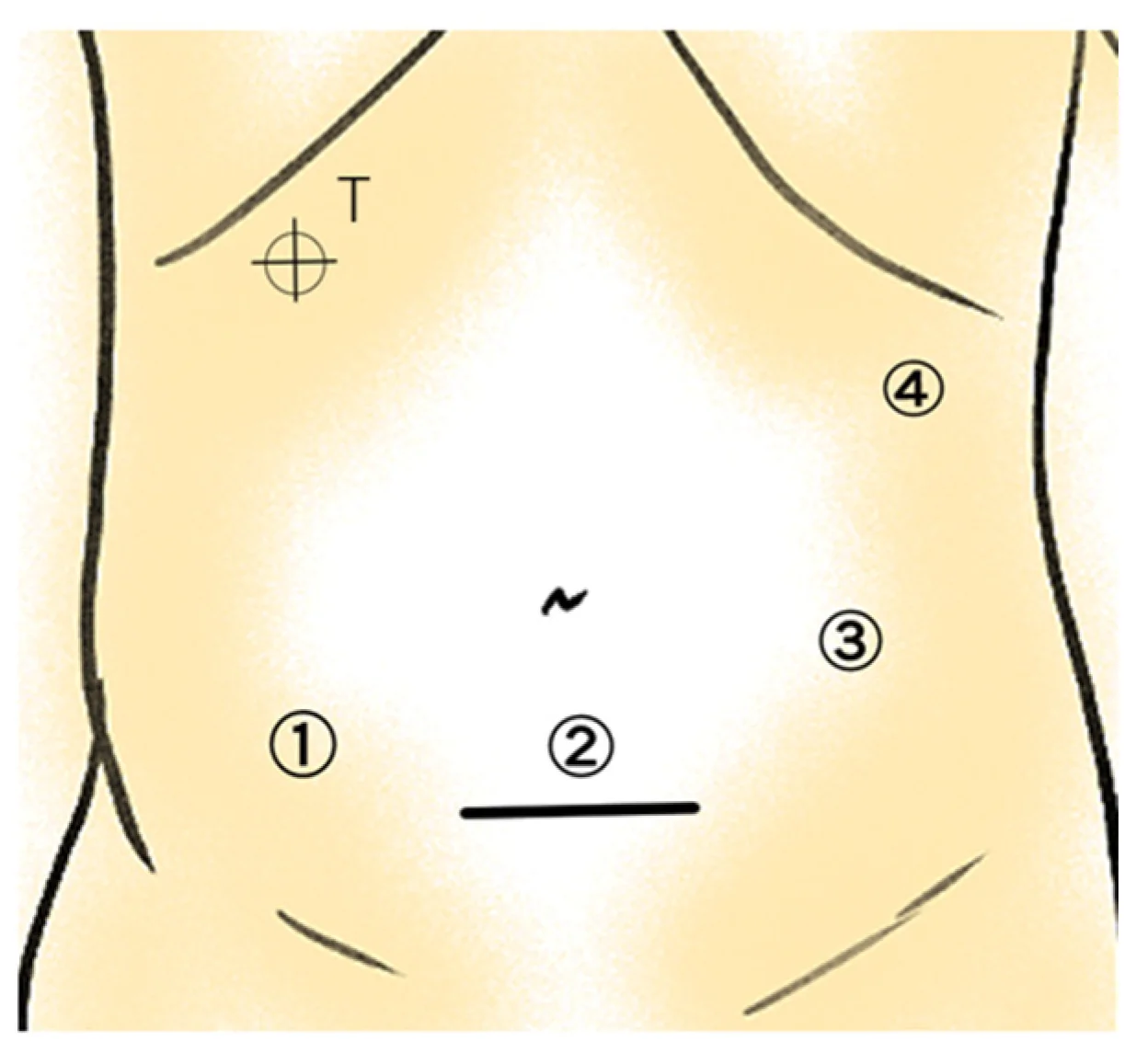
**Port placement for robotic right hemicolectomy.** T indicates the targeting point. Port ①: fenestrated bipolar forceps; Port ②: 30° 3D endoscope; Port ③: monopolar curved scissors; Port ④: tip-up fenestrated grasper. The solid line indicates a 4 cm Pfannenstiel incision.

Under general anesthesia, the patient was placed in the supine position with the legs apart. A 4 cm Pfannenstiel incision was created, and an Alexis^®^ wound retractor (S size; Applied Medical Japan, Tokyo, Japan) was inserted. Pneumoperitoneum was established at 10 mmHg. Three 8 mm robotic trocars and a 12 mm assistant port were placed at approximately 8 cm intervals (Figure 1). The patient was positioned in Trendelenburg with a 5° right-side-up tilt.

**Figure 2:**
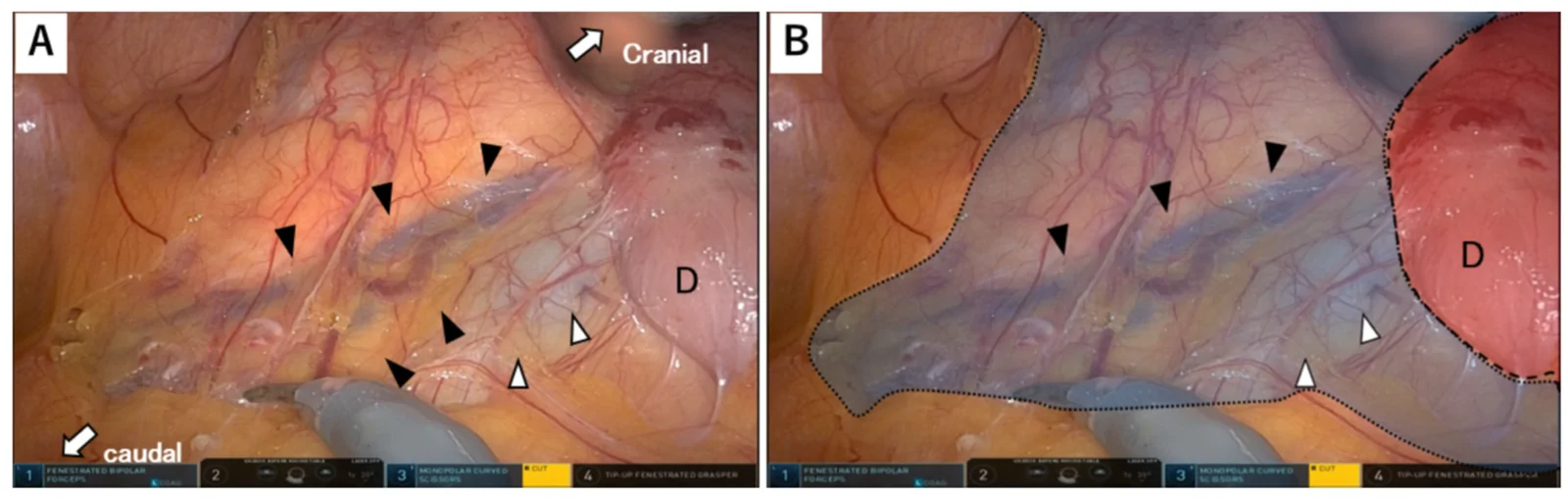
**Dissection plane after the lateral superior approach.** (**A**) Intraoperative view. (**B**) Schematic illustration. D indicates the duodenum. Black arrowheads indicate the right gonadal vessels and ureter within the same embryological fascial plane. The white arrowhead indicates the inferior vena cava. The white arrow indicates the caudal-to-cranial dissection direction. The blue shading indicates the parietal peritoneal fascia, and the red shading indicates the pre-duodeno-pancreatic fascia.

**Figure 3:**
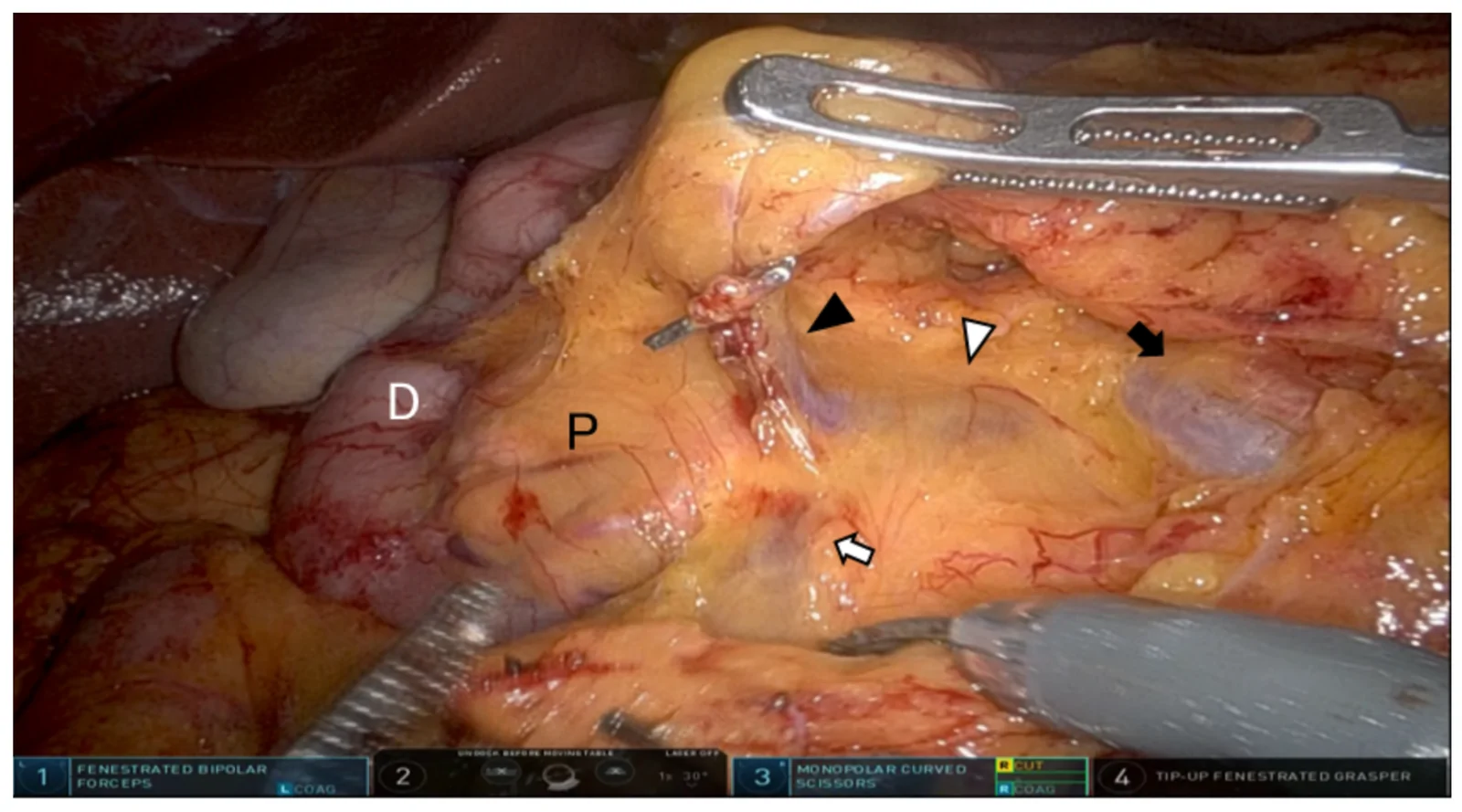
**Exposure of the gastrocolic trunk and superior mesenteric vessels.** D indicates the duodenum. P indicates the pancreas. The black arrowhead indicates the right gastroepiploic vein. The white arrowhead indicates the gastrocolic trunk of Henle. The white arrow indicates the accessory right colic vein. The black arrow indicates the superior mesenteric vessels. These structures are visualized through the intact parietal peritoneal fascia.

The greater omentum was elevated, and the omental sac was entered. Dissection proceeded along the inferior border of the pancreas within the embryological plane between the pancreatic capsule and transverse mesocolon. Lateral mobilization was completed by dividing the gastrocolic and hepatocolic ligaments. The plane along the pre-duodeno-pancreatic fascia and the parietal peritoneal fascia was developed, and the lateral peritoneal attachment (white line of Toldt) was divided. The right ureter, gonadal vessels, and duodenum were identified and preserved (Figure 2). In the LSA, the lateral-to-medial perspective allowed clear identification of the mesocolic fascia, parietal peritoneal fascia, and underlying anterior renal fascia. The dissection plane was intentionally selected according to tumor depth. For tumors with T2 or less invasion, dissection was maintained superficially to the parietal peritoneal fascia to preserve the anterior renal fascia and retroperitoneal structures. For T3 or deeper tumors, the plane was intentionally shifted posteriorly toward the anterior renal fascia to secure adequate radial margins while maintaining mesocolic integrity. The gastrocolic trunk of Henle and accessory right colic vein were exposed, and the accessory right colic vein (ARCV) was ligated at its origin (Figure 3). Dissection along the inferior border of the pancreas exposed the superior mesenteric vein (SMV) and the superior mesenteric artery (SMA).

The retroperitoneal approach was initiated at the level of the common iliac artery and continued cranially to the ligament of Treitz. The previously established superior plane facilitated safe identification of the correct embryological layer.

**Figure 4:**
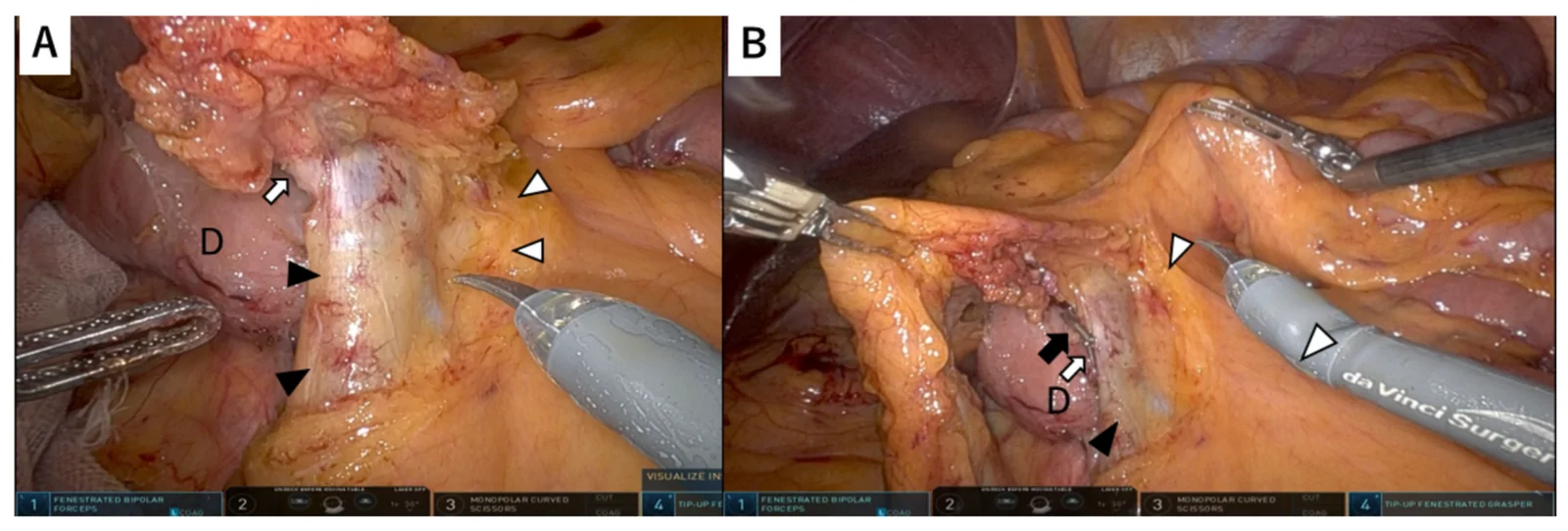
**Exposure of the superior mesenteric vessels and ileocolic dissection.** (**A**) Exposure of the superior mesenteric vessels. (**B**) Lymph node dissection around the ileocolic vessels. D indicates the duodenum. The black arrowhead indicates the superior mesenteric vein. The white arrowhead indicates the superior mesenteric artery and medial boundary of dissection. The white arrow indicates the ligated ileocolic artery. The black arrow indicates the ligated ileocolic vein.

**Figure 5:**
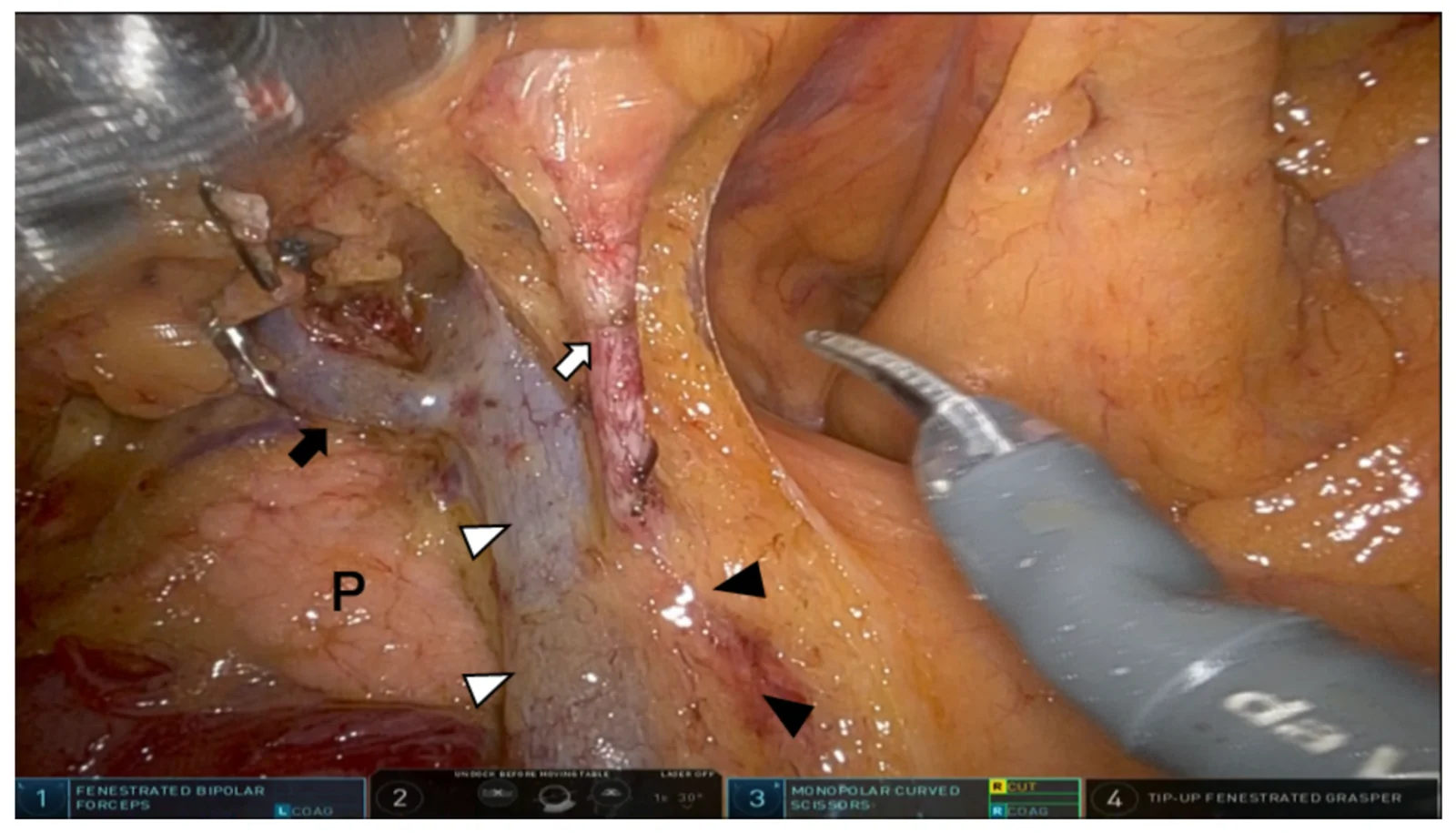
**Middle colic artery exposure and perivascular dissection.** P indicates the pancreas. The black arrowhead indicates the superior mesenteric artery. The white arrowhead indicates the superior mesenteric vein. The white arrow indicates the middle colic artery. The black arrow indicates the gastrocolic trunk of Henle. The right branch of the middle colic artery was ligated at its origin.

Central lymph node dissection was initiated at the ileocolic vessels. The ileocolic artery (ICA) and vein (ICV) were ligated at their origins to accomplish central vascular ligation (CVL) (Figure 4). Dissection was extended cranially toward the SMV. In patients with T3/T4 tumors or clinical stage III disease, the medial boundary of D3 lymphadenectomy was extended beyond the superior mesenteric vein to the left border of the SMA. When present, the right colic artery was divided at its origin. The anatomical relationship between the ICA and ICV varies among patients; notably, the SMA runs anterior to the ICV in approximately 69.1% of cases [17]. Lymph node dissection was then performed around the root of the middle colic artery, followed by division of its right branch and the middle colic vein at their origins. CME with CVL was achieved while preserving embryological fascial planes (Figure 5).

After mesenteric division to the planned transection lines, bowel perfusion was confirmed using indocyanine green (ICG) fluorescence imaging with a near-infrared (NIR) system. Intracorporeal “side-to-side” or “side-to-end” anastomosis was performed, and the specimen was extracted through the Pfannenstiel incision. All procedures were performed by a surgeon experienced in robotic colorectal surgery.

### 2.4. Perioperative Outcome

The primary endpoint was the feasibility of the LSA, assessed by intraoperative outcomes (operative time, blood loss, conversion rate) and postoperative morbidity. Postoperative complications were graded according to the Clavien–Dindo classification. Secondary endpoints included oncological outcomes, such as the number of harvested lymph nodes and short-term survival outcomes (disease-free survival and overall survival).

### 2.5. Statistical Analysis

Statistical analyses were performed using JMP software version 18 (SAS Institute Inc., Cary, NC, USA). Continuous variables are presented as median (range), and categorical variables as number (%). Overall survival and disease-free survival were calculated among patients who underwent curative-intent resection (stage I–III), excluding stage IV disease.

### 2.6. Ethical Approval

This retrospective study was reviewed and approved by the Ethical Review Board of Shimane University, Faculty of Medicine (Shimane, Japan) (approval number: 20240719-2), and was performed in accordance with the Declaration of Helsinki. The Ethical Review Board waived the requirement for informed consent due to the study’s retrospective nature. This study was reported in accordance with the STROBE guidelines.

## 3. Results

A total of 36 patients with right-sided colon cancer underwent robot-assisted right hemicolectomy. The patient characteristics are summarized in Table 1. The median age was 74 years (range 24–93). The cohort consisted of 12 men and 24 women. Regarding physical status, 28 patients (77.8%) were classified as ASA II and 8 (22.2%) as ASA III. The median body mass index was 22.5 kg/m^2^ (range 15.4–26.4). The median hemoglobin level was 11.9 g/dL (range 8.2–15.9), and the median serum albumin level was 3.9 g/dL (range 2.9–5.5), indicating that a subset of patients presented with preoperative anemia and hypoalbuminemia. The median prognostic nutritional index (PNI) was 47.3 (range 34.5–62.3), and the median controlling nutritional status (CONUT) score was 1 (range 0–5), suggesting generally preserved but heterogeneous nutritional status within the cohort. The median Charlson Comorbidity Index was 3 (range 2–9). The tumor location was the cecum in 8 patients (22.2%), ascending colon in 24 (66.7%), and transverse colon in 4 (11.1%).

The pathological findings are shown in Table 2. Histologically, 31 tumors (86.1%) were well or moderately differentiated adenocarcinoma, 1 (2.8%) was poorly differentiated adenocarcinoma, and 4 (11.1%) were mucinous adenocarcinoma. According to the TNM classification, T1/2/3/4 tumors were observed in 8 (22.2%), 4 (11.1%), 16 (44.4%), and 8 (22.2%) patients, respectively. N1/2/3 status was identified in 3 (8.3%), 5 (13.9%), and 3 (8.3%) patients, respectively. The pathological stage was I in 12 patients (33.3%), II in 11 (30.6%), III in 9 (25.0%), and IV in 4 (11.1%). The median number of harvested lymph nodes was 31 (range 15–63). Lymph node metastasis was present in 11 patients (30.6%), with a median of 6 positive nodes (range 4–23). Vascular and lymphatic invasion were each observed in 10 patients (27.8%). The median number of harvested main lymph nodes around the middle colic artery (No. 223 according to the Japanese Classification of Colorectal Carcinoma) was 4 (range 0–17), and metastasis in this region was identified in 3 patients, with a median of 5 positive nodes (range 1–15).

Intraoperative and postoperative outcomes are summarized in Table 3. No intraoperative complications or conversion to open surgery occurred. The median operative time was 289 min (range 174–489), including a median console time of 188 min (range 86–421). The median blood loss was 10 mL (range 5–220). One patient required an intraoperative blood transfusion because of pre-existing anemia.

Postoperative complications included ileus in 4 patients (11.1%), severe diarrhea in 1 (2.8%), and deep vein thrombosis in 1 (2.8%). All complications were Clavien–Dindo grade II or lower and were managed conservatively. No lymphatic leakage was observed. The median time to resumption of oral intake was 5 days (range 3–10), and the median postoperative hospital stay was 11 days (range 6–33).

**Figure 6:**
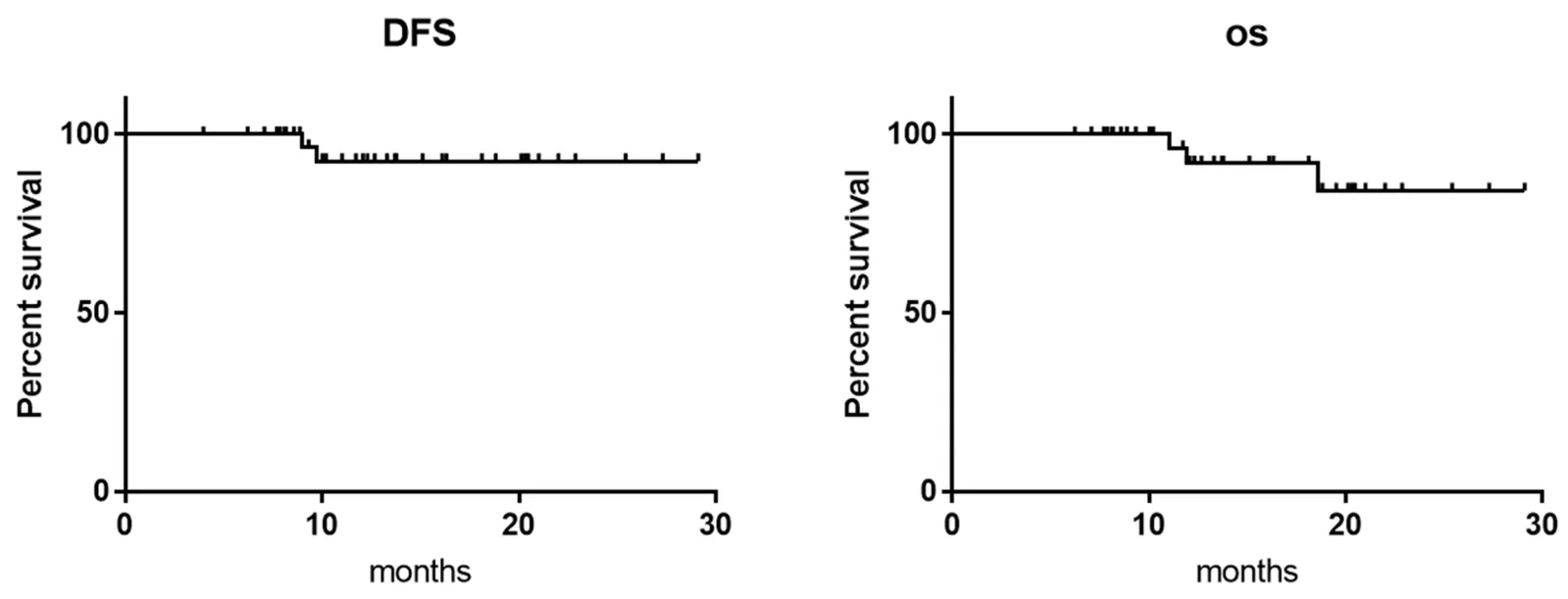
**Kaplan–Meier curves.** The estimated 2-year disease-free survival and overall survival rates were 92.4% and 84.2%, respectively. Dotted lines indicate the 95% confidence intervals. Abbreviations: DFS, disease-free survival; OS, overall survival.

Among patients who underwent curative-intent resection (stage I–III), the median follow-up duration was 16.9 months (range, 10.0–31.7). Kaplan–Meier analysis estimated the two-year disease-free survival and overall survival rates at 92.4% and 84.2%, respectively (Figure 6).

## 4. Discussion

Numerous surgical approaches have been described for right-sided colon cancer, reflecting ongoing uncertainty regarding the optimal dissection plane and sequence of mobilization. While lateral approaches were traditionally favored in open surgery because of their anatomical clarity, their application in laparoscopy is technically demanding due to restricted visualization and limited instrument mobility. Consequently, medial-to-lateral and retroperitoneal approaches have been widely adopted in laparoscopic right hemicolectomy [13,14,15]. Robotic surgery overcomes many of these limitations by enabling stable multidirectional tissue traction and precise dissection using articulated instruments. In this study, we demonstrated that a lateral superior approach can be safely performed in robotic right hemicolectomy, allowing secure CME and D3 lymphadenectomy. The standardized sequence of dissection and early identification of embryological landmarks may facilitate procedural standardization among robotic colorectal surgeons. A practical advantage of the LSA is the early identification of the inferior border of the pancreas and the gastrocolic trunk from a superior perspective. This facilitates the safe exposure and management of critical vascular structures, including the accessory right colic vein, before central lymph node dissection. This standardized sequence may facilitate a standardized surgical workflow during robotic right hemicolectomy.

Complete mesocolic excision (CME) is based on embryological principles; however, the optimal dissection plane for right-sided colon cancer remains controversial because the retrocolic fascia comprises multiple embryological layers and anatomical terminology has been used inconsistently across studies [6,18,19,20,21].

**Figure 7:**
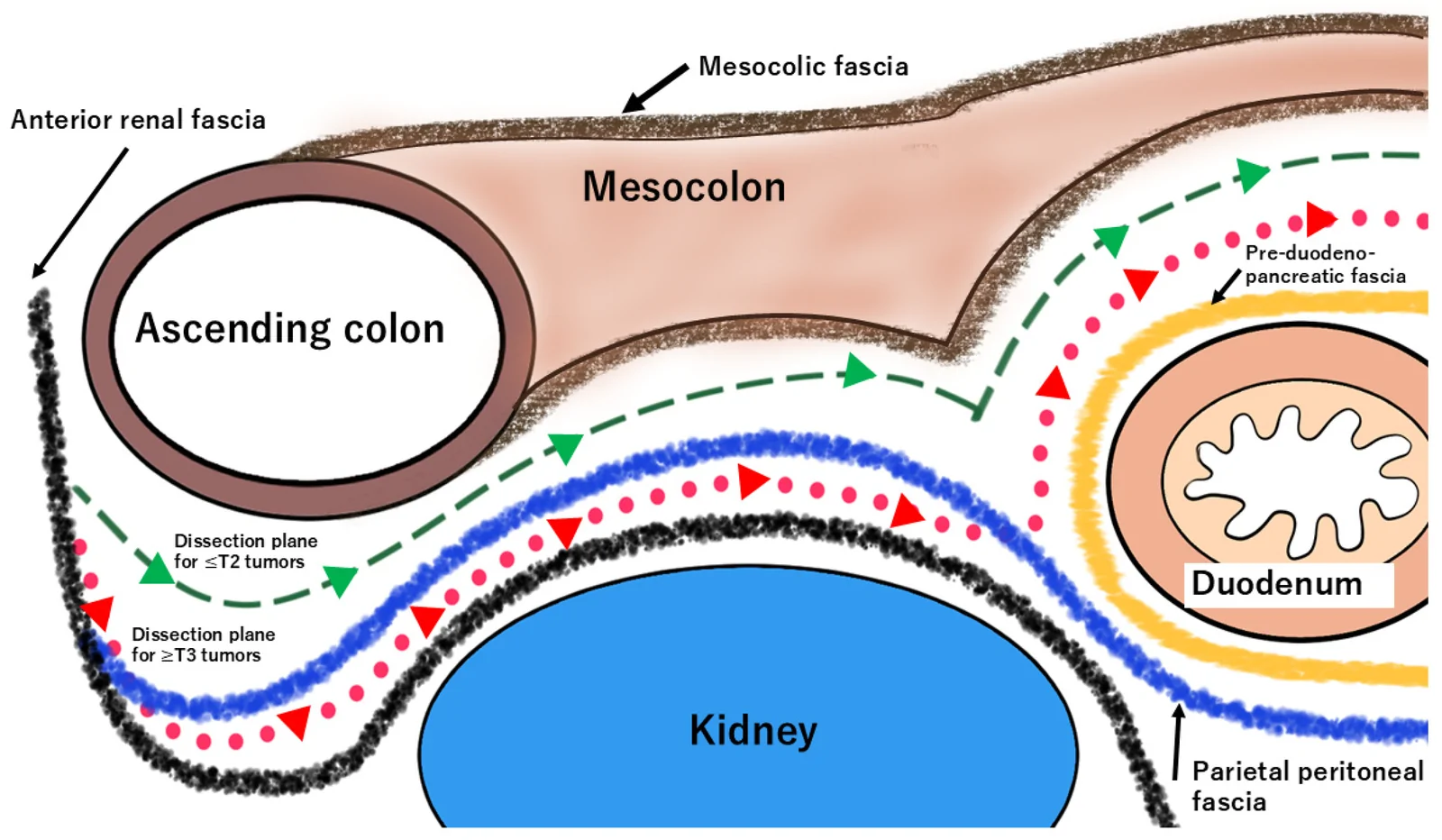
**Layer-oriented dissection strategy in the lateral superior approach.** The LSA provides a lateral-to-medial perspective that facilitates identification of the mesocolic fascia, parietal peritoneal fascia, anterior renal fascia, and pre-duodeno-pancreatic fascia. For tumors with ≤T2 invasion, dissection is performed superficial to the parietal peritoneal fascia, preserving retroperitoneal structures (green dashed line). For ≥T3 tumors, the plane can be shifted posteriorly toward the anterior renal fascia to secure adequate radial margins (red dotted line). This strategy enables depth-tailored selection of the optimal dissection plane.

Wedel et al. demonstrated that the retrocolic fascia comprises several distinct embryological fascial layers and proposed standardized anatomical planes for complete mesocolic excision of the right colon [22]. Based on these anatomical findings, the optimal dissection plane may be selected according to tumor depth. For tumors with ≤T2 invasion, dissection is performed superficially to the parietal peritoneal fascia, thereby preserving the anterior renal fascia and retroperitoneal structures. In contrast, for ≥T3 tumors, the dissection plane can be extended posteriorly toward the anterior renal fascia to obtain an adequate radial margin (Figure 7). This concept appears anatomically consistent with the principle of complete mesocolic excision, in which preservation of an intact mesocolic envelope and, when oncologically indicated, extension to a deeper embryological plane may contribute to securing an adequate radial margin while avoiding unnecessary retroperitoneal dissection [22,23,24,25]. Our lateral superior approach facilitates the clear identification of these embryological fascial layers, enabling tailored plane selection while preserving mesocolic integrity. Unlike conventional medial approaches, the LSA first establishes the cranial dissection plane along the inferior border of the pancreas, providing an anatomical landmark for subsequent lymph node dissection and vascular control. This anatomical orientation may enable intentional selection of the optimal embryological dissection plane according to tumor depth, rather than relying on a fixed surgical plane. Such flexibility may facilitate anatomically rational plane selection. These technical advantages may facilitate dissection parallel to embryological fascial planes.

Previous robotic CME series have consistently demonstrated the safety and feasibility of robotic right hemicolectomy, with low conversion rates, acceptable postoperative morbidity, and adequate lymph node harvest [26,27]. The perioperative outcomes in the present study were comparable to those reported in the literature, with no intraoperative complications or conversions, a median blood loss of 10 mL, a postoperative complication rate of 16.7%, and a median lymph node harvest of 31.

However, unlike previous studies that primarily focused on establishing the feasibility of robotic CME, our study introduces a layer-oriented strategy that enables selection of the embryological dissection plane according to the depth of tumor invasion. Rather than applying a uniform dissection plane for all patients, the proposed lateral superior approach enables selective dissection within the appropriate fascial layer while preserving the principles of complete mesocolic excision. In addition, the magnified three-dimensional view and articulated instrumentation provided by the robotic platform may facilitate accurate identification of tissue planes and precise dissection along the intended embryological layer, thereby supporting standardized execution of the proposed layer-oriented approach.

Another unresolved issue in right-sided colon cancer surgery is the medial margin of D3 lymph node dissection. In the East, the SMV is commonly regarded as the medial boundary, and the JSCCR recommends skeletonization of the SMV with division of the colonic artery to its left side [12]. In contrast, recent Western studies advocate SMA-based dissection, citing metastatic distribution and embryological considerations [17,28]. Although SMA-oriented dissection may improve oncological clearance, it has been reported to be associated with an increased risk of chylous leakage, underscoring the need for individualized strategies. In our approach, early superior identification of both the SMA and SMV allows intraoperative selection of the appropriate medial margin. In this series, SMA-based lymphadenectomy was performed without major vascular injury or chylous leakage, supporting the feasibility and safety of this strategy. Although all procedures were performed by an experienced robotic colorectal surgeon, a potential learning curve effect cannot be excluded.

Adequate lymph node harvest is associated with improved oncological outcomes [29]. The median number of retrieved lymph nodes was 31, and central lymph node metastasis around the middle colic artery was identified in 3 cases (median of 4 metastatic nodes), supporting the technical feasibility and oncological soundness of the procedure. Postoperative morbidity was low, and short-term survival outcomes were comparable to those reported in previous studies. However, longer follow-up is necessary to validate the long-term oncological benefit of this approach.

### Limitations

This study has several limitations. First, its retrospective single-center design, relatively small sample size, and absence of a comparative cohort introduce potential selection bias, limit the statistical power of the analysis, and preclude definitive conclusions regarding the superiority of this approach over established robotic techniques. Second, all procedures were performed by a single experienced robotic colorectal surgeon, which may limit the generalizability and reproducibility of the findings in other institutions or among surgeons with different levels of robotic experience. Third, the median follow-up period was relatively short, precluding the definitive assessment of long-term oncological outcomes. Finally, the concordance between preoperative and pathological T stage was not formally evaluated. Because pathological T stage is unavailable intraoperatively, layer selection was based on the preoperative clinical assessment of tumor depth and subsequently verified by pathological examination. Therefore, discrepancies between clinical and pathological T stage may have influenced plane selection in some patients. Future prospective multicenter studies should evaluate the accuracy of preoperative tumor staging and its influence on optimal layer selection.

## 5. Conclusions

The lateral superior approach facilitates the early identification of embryological planes and intentional selection of the optimal dissection layer according to tumor depth while maintaining the principles of CME and CVL. The present findings support the feasibility and safety of this layer-oriented approach and suggest the potential value of a layer-oriented strategy for the tumor depth-tailored selection of the embryological dissection plane. Further multicenter comparative studies with longer follow-up are warranted to validate its generalizability and long-term oncological outcomes.

## Figures and Tables

**Table 1:** Patients’ preoperative characteristics.

Variables		No. Patients (*n* = 36)	
Age (year) *		74	[24–93]
Sex	male	12	33.3%
	female	24	66.7%
BMI (kg/m^2^) *		22.5	[15.4–26.4]
ASA-PS	I	0	
	II	28	77.8%
	III	8	22.2%
Tumor locations	cecum	8	22.2%
	ascending colon	24	66.7%
	right side of transverse colon	4	11.1%
Hb (g/dL)		11.9	[8.2–15.9]
Alb (g/dL)		3.9	[2.9–5.5]
Charlson comorbidity index		3	[2–9]
PNI		47.3	[34.5–62.3]
CONUT score		1	[0–5]

* Data are expressed as a median (range). Abbreviations: BMI, body mass index; ASA-PS, American Society of Anesthesiologists Physical Status; PNI, prognostic nutritional index; CONUT, Controlling Nutritional Status.

**Table 2:** Pathological characteristics.

Variables		Superior Approach	
Tumor size (mm) *		35	[10–110]
Histological differentiation	Poorly differentiated	1	2.8%
	Moderately differentiated	15	41.7%
	Well differentiated	16	44.4%
	Mucinous adenocarcinoma	4	11.1%
Pathological T stage	T1	8	22.2%
	T2	4	11.1%
	T3	16	44.4%
	T4	8	22.2%
Pathological N stage	N0	25	69.4%
	N1	3	8.3%
	N2	5	13.9%
	N3	3	8.3%
Pathological M stage	M0	32	88.9%
	M1	4	11.1%
Stage	I	12	33.3%
	II	11	30.6%
	III	9	25.0%
	IV	4	11.1%
Total harvested lymph nodes		31	[15–63]
Number of positive lymph nodes *		6	[4–23]
Harvested No. 223 lymph nodes		4	[0–17]
Patients with positive No. 223 lymph nodes		3	8.3%
Lymphovascular invasion	Yes	10	27.8%
	No	26	72.2%
Perineural invasion	Yes	10	27.8%
	No	26	72.2%

* Data are presented as median (range).

**Table 3:** Intraoperative and postoperative outcomes.

Variables			Superior Approach	
Operative time (min)			289	[174–489]
Console time (min)			188	[86–421]
Intraoperative blood loss (mL)			10	[5–220]
Intraoperative transfusion	Yes		1	2.7%
	No		35	97.2%
Intraoperative complications	Yes		0	
	No		36	100.0%
Postoperative hospital stay (days)			11	[6–33]
Time to first diet (days)			5	[3–10]
Postoperative complications	Yes		6	16.7%
		ileus	4	11.1%
		diarrhea	1	2.8%
		DVT	1	2.8%
	No		30	83.3%
Classification of complication (Clavien–Dindo system)	I–II		6	16.7%
	III–IV		0	

Abbreviations: DVT, deep vein thrombosis.

## Data Availability

The datasets generated and/or analyzed during the current study are not publicly available because they contain information that could compromise the privacy of research participants but are available from the corresponding author on reasonable request, subject to approval by the institutional ethics committee.

## Abbreviations

CME, complete mesocolic excision; LSA, lateral superior approach; CVL, central vascular ligation; TNM, Tumor–Node–Metastasis classification; CT, computed tomography; SMV, superior mesenteric vein; SMA, superior mesenteric artery; PNI, prognostic nutritional index; CONUT, controlling nutritional status.
